# Survival Benefit of Kidney Transplantation in Patients With End-Stage Kidney Disease and Prior Acute Myocardial Infarction

**DOI:** 10.3389/ti.2023.11491

**Published:** 2023-08-24

**Authors:** Deok-Gie Kim, Dong-Hyuk Cho, Kihyun Kim, Sung Hwa Kim, Juhan Lee, Kyu Ha Huh, Myoung Soo Kim, Dae Ryong Kang, Jae Won Yang, Byoung Geun Han, Jun Young Lee

**Affiliations:** ^1^ Department of Surgery, Yonsei University College of Medicine, Seoul, Republic of Korea; ^2^ Department of Cardiology, Yonsei University Wonju College of Medicine, Wonju, Republic of Korea; ^3^ Department of Cardiology, Gangneung Dong-in Hospital, Gangneung, Republic of Korea; ^4^ National Health Big Data Clinical Research Institute, Yonsei University Wonju College of Medicine, Wonju, Republic of Korea; ^5^ Department of Biostatistics, Yonsei University Wonju College of Medicine, Wonju, Republic of Korea; ^6^ Department of Precision Medicine, Yonsei University Wonju College of Medicine, Wonju, Republic of Korea; ^7^ Department of Nephrology, Yonsei University Wonju College of Medicine, Wonju, Republic of Korea

**Keywords:** mortality, acute myocardial infarction, end stage kidney disease (ESKD), kidney transplantation (KT), major adverse cardiovascular events (MACE)

## Abstract

Patients with end stage kidney disease (ESKD) and a previous acute myocardial infarction (AMI) have less access to KT. Data on ESKD patients with an AMI history who underwent first KT or dialysis between January 2007 and December 2018 were extracted from the Korean National Health Insurance Service. Patients who underwent KT (*n* = 423) were chronologically matched in a 1:3 ratio with those maintained on dialysis (*n* = 1,269) at the corresponding dates, based on time-conditional propensity scores. The 1, 5, and 10 years cumulative incidences for all-cause mortality were 12.6%, 39.1%, and 60.1% in the dialysis group and 3.1%, 7.2%, and 14.5% in the KT group. Adjusted hazard ratios (HRs) of KT versus dialysis were 0.17 (95% confidence interval [CI], 0.12–0.24; *p <* 0.001) for mortality and 0.38 (95% CI, 0.23–0.51; *p <* 0.001) for major adverse cardiovascular events (MACE). Of the MACE components, KT was most protective against cardiovascular death (HR, 0.23; 95% CI, 0.12–0.42; *p <* 0.001). Protective effects of KT for all-cause mortality and MACE were consistent across various subgroups, including patients at higher risk (e.g., age >65 years, recent AMI [<6 months], congestive heart failure). KT is associated with lower all-cause mortality and MACE than maintenance dialysis patients with a prior AMI.

## Graphical Abstract

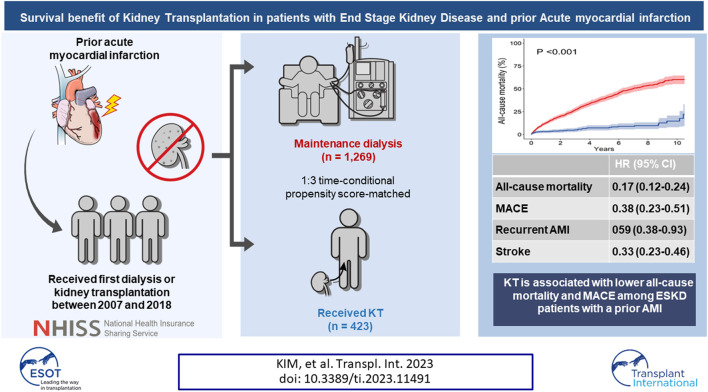

## Introduction

Cardiovascular disease (CVD) is the most common cause of death in patients with end-stage kidney disease (ESKD) [1]. For patients with ESKD requiring renal replacement therapy, kidney transplantation (KT) is the best treatment option to reduce the risk of CVD [2]. However, approximately 50% of patients with ESKD already have CVD before initiating renal replacement therapy [3]. Furthermore, the number of KT candidates with a history of CVD is gradually increasing because of the increasing number of KT candidates who are older or who have waited for an extended period of time for a deceased donor kidney [4]. Prior CVD history is the strongest risk factor for posttransplant coronary artery disease [5, 6] and affects physicians’ decisions regarding whether to proceed with KT.

The Kidney Disease Improving Global Outcome guidelines suggest that patients with ESKD who have CVD can be candidates for KT after appropriate cardiologic evaluation [7]. However, in the real world, patients with CVD have low access to KT, as reported in a French registry study [8]. A United States (US) registry study demonstrated that underlying CVD was more frequent in patients who were not informed about KT than in those who were informed [9]. Furthermore, in a recent Australian study, patients with CVD were half as likely to be waitlisted for deceased donor KT or to undergo living-donor KT, compared with individuals without CVD [10]. This low access to KT may be attributed to both patients and physicians assuming that the comorbid CVD can lead to poorer outcomes from KT than remaining on dialysis; however, the validity of this assumption has not been well investigated.

Among CVD events, acute myocardial infarction (AMI) is one of the strongest risk factors for mortality in patients with ESKD [11]. According to the US Renal Data System report, mortality after AMI in patients with chronic kidney disease stages 4 or 5 was more than 50% after 2 years [12]. To our knowledge, only one study including patients with a prior AMI has compared survival between patients treated with KT and those treated with dialysis. Using an Argentina registry [13], this study showed a survival benefit with KT in patients older than 60 years and with multiple comorbidities. However, less than 20% of patients included in the analyses had a previous AMI, emphasizing the need for more studies to inform physician decisions about KT in patients with prior AMI. Moreover, AMI is distinct from other comorbidities when considering KT because of the possibility for postoperative acute CVD events, as well as the risk of bleeding associated with potent antiplatelet agents [14, 15].

To determine an optimal treatment strategy for patients with ESKD who have a history of AMI, it is necessary to compare major adverse cardiovascular events (MACE) and mortality after KT with the same outcomes in patients remaining on dialysis. Therefore, we used a nationwide database to compare the survival benefit of KT with that of maintenance dialysis in patients with ESKD and a prior AMI.

## Patients and Methods

### Data Sources

The Korean government uses the National Health Insurance Service (NHIS) database, which covers 97% of all citizens (almost 50 million people) in the Republic of Korea. All hospitals in Korea send information about inpatient and outpatient visitations, procedures, prescriptions, and national health examination data to the NHIS. The NHIS then assigns diagnosis codes based on the International Classification of Disease (ICD), 10th edition. These data resources are widely validated and used for epidemiologic studies [16]. The NHIS provides information from claims data for research purposes and includes mortality records with the cause and date of death, which are retrieved from the Statistics Korea database^1^. Data are available with the approval and oversight of the NHIS (NHIS-2019-1-448) through the Korean National Health Insurance Sharing Service^2^. The specific codes used to define every diagnosis, procedure, and drug in this study are shown in Supplementary Tables S1, S2.

### Study Population

This study used NHIS data of patients newly diagnosed with ESKD (defined as requiring hemodialysis, peritoneal dialysis, and/or KT) between January 2007 and December 2018. As KT was usually performed after a period of dialysis treatment (except for cases of pre-emptive KT), comparing KT and dialysis based on the date of ESKD diagnosis would inevitably lead to immortal time bias of patients receiving KT, thereby resulting in an overestimation of the survival of these patients [17]. To minimize this bias, we applied a “prevalent new user design,” which has been used in pharmacoepidemiology. Treating ESKD as a “disease,” dialysis as a “former drug,” and KT as a “new drug,” in accordance with the components of a prevalent new user design, we established separate time-based exposure cohorts for dialysis and KT [18, 19]. The time interval of ±3 months surrounding the date of KT was used to select the dialysis control patients (Supplementary Figure S1). The cohort entry date was defined as the KT date for the KT cohort and the corresponding date of dialysis prescription for the dialysis cohort. When patients included in the dialysis cohort at certain cohort entry dates subsequently underwent KT, they were censored and reused as KT subjects based on the date of KT. This provides an intention-to-treat approach for comparing the effects of proceeding with KT versus continuing on dialysis alone or waiting for further KT at the given entry date. Baseline characteristics, including prior AMI and exclusion criteria, were based on the cohort entry date of each subject. Prior AMI was defined as the first diagnosis of AMI with a hospital admission duration of >2 days.

In this study, we included only patients with a prior AMI within 5 years before each cohort entry date. We excluded patients who were <19 or >75 years of age at the time of cohort entry. Patients diagnosed with cancer (because of its effects on KT eligibility) and those diagnosed with stroke, valvular heart disease, and/or cardiac conduction abnormality (because of the effects of these non-AMI CVDs on KT accessibility and outcomes) within 5 years before cohort entry were also excluded. In addition, patients receiving dialysis for >10 years before KT were excluded to eliminate individuals in excellent medical condition while on dialysis, who then received KT.

### Matching

The KT and dialysis cohorts were matched according to these steps: 1) the dialysis date corresponding to the KT date was set as the cohort entry date in the dialysis cohort, 2) exclusion criteria were applied based on the cohort entry date, 3) only patients with an AMI within 5 years before cohort entry were selected, and 4) dialysis patients were matched to KT patients based on time-conditional propensity scores calculated using conditional logistic regression stratified by dialysis cohort or KT cohort [20]. The covariates used for generating the time-conditional propensity scores were age, sex, diabetes mellitus, calendar year of ESKD diagnosis, calendar year of cohort entry date, interval from ESKD to cohort entry date, interval from AMI to cohort entry date, type of AMI treatment (percutaneous coronary intervention [PCI], coronary artery bypass graft [CABG], or medication only), and secondary prevention drugs after AMI (angiotensin converting enzyme inhibitors or angiotensin receptor blockers, beta blockers, statins, antiplatelet agents (aspirin, clopidogrel, cilostazole, ticlopidine, prasugrel, ticagrelor, or triflusal) or calcium channel blockers). Use of a drug was defined as being prescribed the drug >2 times during outpatient visits within 1 year before cohort entry.

Patients with underlying conditions were matched according to the Charlson Comorbidity Index (CCI) calculated using data from the 5 years period before cohort entry [13, 14]. Diabetes mellitus and congestive heart failure (CHF) were matched separately from CCI because of their prominent effects on CVD and survival in patients with ESKD. Matching was performed with a 1:3 ratio, without replacement, and in chronological order. If a matched dialysis subject underwent KT during follow-up, the patient was censored at the time of KT, then included in the KT cohort and matched with other patients in the dialysis cohort based on the newly designated entry date (KT date).

### Outcomes

The primary outcome of this study was all-cause mortality and MACE, which was a composite of cardiovascular mortality, recurrent AMI, and stroke. The secondary outcomes were each component of MACE and a coronary revascularization procedure (PCI or CABG). Cardiovascular mortality was defined as any death with an ICD-10 code of I00–I99, as confirmed in the Statistics Korea database. Recurrent AMI was defined as hospitalization for the AMI diagnosis code and/or coronary revascularization. The study population was followed from each cohort entry date until the date of death, 31 December 2018, or the date of subsequent KT (in the dialysis cohort), whichever came first.

### Statistical Analysis

Matching on time-conditional propensity scores was performed with greedy (nearest neighbor) matching techniques [21]. Covariate balances were considered adequate when standardized mean differences after matching were <0.1 [22]. Baseline characteristics were compared between the KT and matched dialysis groups using the t-test or chi-squared test, as appropriate. Continuous variables were expressed as mean ± standard deviation, and categorical variables were expressed as number (percentage). Kaplan–Meier survival curves with the log-rank test were used to compare cumulative outcome incidences. Hazard ratios for each outcome were obtained before and after being adjusted for baseline characteristics using Cox proportional-hazard regression analysis. Death from causes other than CVD and loss to follow-up were considered as competing risks when comparing MACE and each component. Moreover, regression analyses were performed by Fine and Gray’s model for those outcomes. Sensitivity analyses were performed in various subgroups for all-cause mortality and MACE: age (<65 vs. ≥65 years), sex, AMI treatment method (PCI/CABG vs. medication alone), interval from AMI to cohort entry date (<6 vs. 6–12 vs. ≥12 months), year of cohort entry date (2007–2012 vs. 2013–2018), interval from ESKD to cohort entry date (<1 vs. 2–5 vs. 5–10 years), CCI (<9 vs. ≥9), and CHF (presence or absence). The sensitivity of the effect of KT was analyzed by creating an interaction of the *p*-value between KT versus non-KT and each subgroup. Furthermore, to confirm whether KT adversely affected outcomes during the early post-KT period, we performed several independent analyses (<3, <6, and <12 months after cohort entry), where administrative censoring was applied to the maximum time point (or earlier if the patient was lost to follow-up).

All *p* values were two-sided, and *p* values <0.05 were considered significant. Analyses were performed using the statistical package SAS 9.4 (SAS Institute, Cary, NC, United States) and R version 4.2.0 for Windows^3^.

The Institutional Review Board (IRB) of the Yonsei University Wonju College of Medicine (Wonju, Korea) approved this study (IRB number: CR319308). Informed consent was waived because anonymous and de-identified information was used for the analyses. This trial was registered with the Clinical Research Information Service, Republic of Korea (KCT0005759).

## Results

### Patient Characteristics

**FIGURE 1:**
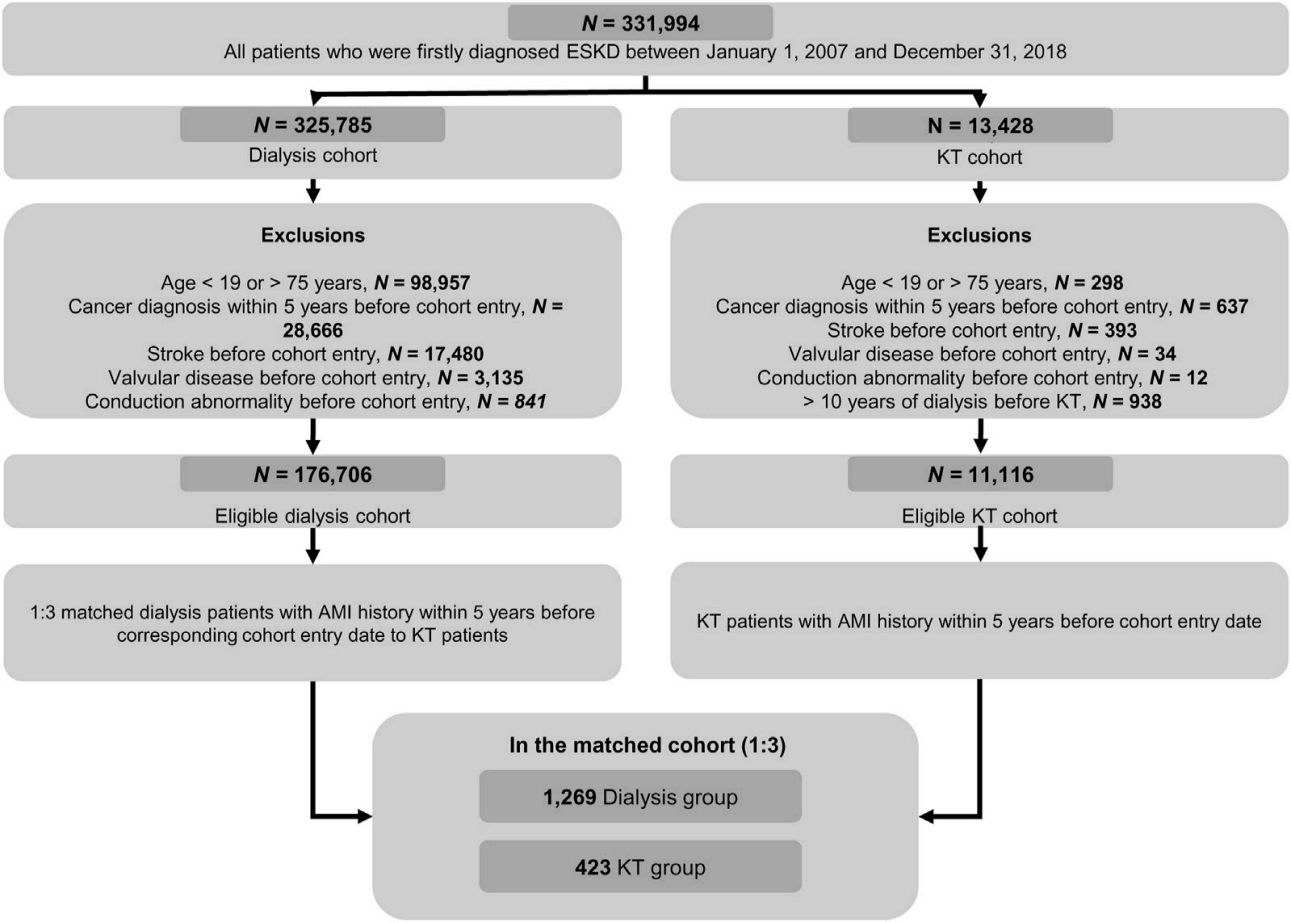
Flow diagram showing selection of the study population from the Korean National Health Insurance Service Database. AMI, acute myocardial infarction; ESKD, end-stage kidney disease; KT, kidney transplantation.

Of the 331,994 first diagnosed with ESKD during the study period, 325,785 were in the dialysis cohort and 13,428 were in the KT cohort (Figure 1). From these, a 1:3 matched ESKD population with prior AMI were included in the comparative analyses: 1,269 dialysis patients were matched to 423 KT patients based on time-conditional propensity scores with appropriate balance (Supplementary Figure S2). Baseline characteristics are shown in Table 1. The mean age was 52.3 ± 10.8 years for the dialysis group and 53.3 ± 11.1 years for the KT group (*p* = 0.979). Men were more frequent in both groups (73.8% in the dialysis group vs. 73.5% in the KT group; *p* = 0.924). Year of first ESKD diagnosis and cohort entry date were similar between the two groups. As a result of chronologic matching, the interval from ESKD diagnosis to cohort entry date was similar between the two groups, not only when stratified by <1 year, 1–5 years and 5–10 years, but also when mean values were compared (30.6 ± 29.1 months vs. 33.1 ± 29.7 months; *p* = 0.482). Mean interval from AMI to cohort entry date was 25.0 ± 18.1 months in the dialysis group and 23.9 ± 18.8 months in the KT group. Additionally, the two groups had similar treatment modalities for their prior AMI (CABG [7.0% vs. 8.0%], PCI [44.7% vs. 40.0%], and medical treatment alone [48.3% vs. 52.0%]; *p* = 0.226), which were consistent with AMI treatment distributions previously reported in patients with chronic kidney disease [23, 24]. Secondary prevention drugs after AMI were used in similar percentages of patients at cohort entry in both groups. The mean CCI value was more than 8 and similar in both groups (8.7 ± 2.6 vs. 8.6 ± 2.4; *p* = 0.600). The frequencies of each CCI component were similar between groups, except peripheral vascular disease, dementia, and hemi- or paraplegia.

**TABLE 1:** Baseline characteristics between matched ESKD patients with AMI history.

Variables	Dialysis (*n* = 1,269)	KT (*n* = 423)	*P*
Age	52.3 ± 10.8	53.3 ± 11.1	0.979
Sex, male	936 (73.8)	311 (73.5)	0.924
Year of first ESKD diagnosis			0.188
2007–2012	906 (71.39)	316 (74.7)	
2013–2018	363 (28.61)	107 (25.3)	
Year of cohort entry date			0.998
2007–2012	471 (37.12)	157 (37.12)	
2013–2018	798 (62.88)	266 (62.88)	
Interval from ESKD to cohort entry date			0.999
<1 year	555 (43.7)	185 (43.7)	
1–5 years	525 (41.4)	175 (41.4)	
5–10 years	189 (14.9)	63 (14.9)	
Mean, month	30.6 ± 29.1	33.1 ± 29.7	0.482
Interval from AMI to cohort entry date, months	25.0 ± 18.1	23.9 ± 18.8	0.257
AMI treatment			0.226
CABG	89 (7.0)	34 (8.0)	
PCI	567 (44.7)	169 (40.0)	
Medical treatment	613 (48.3)	220 (52.0)	
Secondary preventive drugs after AMI			
ACEi or ARB	941 (74.2)	313 (74.0)	0.949
Beta blocker	958 (75.5)	319 (75.4)	0.974
Statin	846 (66.7)	265 (62.7)	0.132
Antiplatelet agent	896 (70.6)	287 (67.9)	0.819
Calcium channel blocker	1,124 (88.6)	385 (91.0)	0.284
Charlson Comorbidity Index	8.7 ± 2.6	8.6 ± 2.4	0.600
Diabetes	1,117 (88.0)	375 (88.7)	0.728
Congestive heart failure	761 (60.0)	238 (56.3)	0.180
Peripheral vascular disease	645 (50.8)	184 (43.5)	0.009
Dementia	64 (5.0)	9 (2.1)	0.011
Chronic pulmonary disease	763 (60.1)	249 (58.9)	0.647
Rheumatologic disease	114 (9.0)	46 (10.9)	0.250
Peptic ulcer disease	758 (59.7)	268 (63.4)	0.186
Mild liver disease	713 (56.2)	250 (59.1)	0.294
Moderate or severe liver disease	40 (3.2)	17 (4.0)	0.392
Hemiplegia or paraplegia	54 (4.3)	9 (2.1)	0.045
AIDS	0 (0.0)	2 (0.5)	0.062

Abbreviations: ACEi, angiotensin converting enzyme inhibitors; AIDS, acquired immune deficiency syndrome; AMI, acute myocardial infarction; ARBs, angiotensin receptor blockers; CABG, coronary artery bypass graft; ESKD, end stage kidney disease; KT, kidney transplantation; PCI, percutaneous coronary intervention.

### Details of Kidney Transplantation Recipients

Of the 423 patients who underwent KT, 185 (43.7%) received <1 year of pre-transplant dialysis before KT, including 66 (15.6%) who underwent pre-emptive KT. The median pre-transplant dialysis duration was 29.6 (interquartile range, 9.7–57.4) months. There were 9 (2.1%) in-hospital deaths after KT: 6 were due to recurrent AMI and 3 were from an unknown cause. There were 13 (3.1%) in-hospital MACE, including 6 cardiovascular deaths and 7 cases of coronary artery disease treated with PCI. The cumulative incidences of graft failure (restart of dialysis or re-transplantation) were 2.4%, 5.2%, and 8.9% at 1, 5, and 10 years after KT, respectively (Supplementary Figure S3).

### Primary Outcomes

**FIGURE 2:**
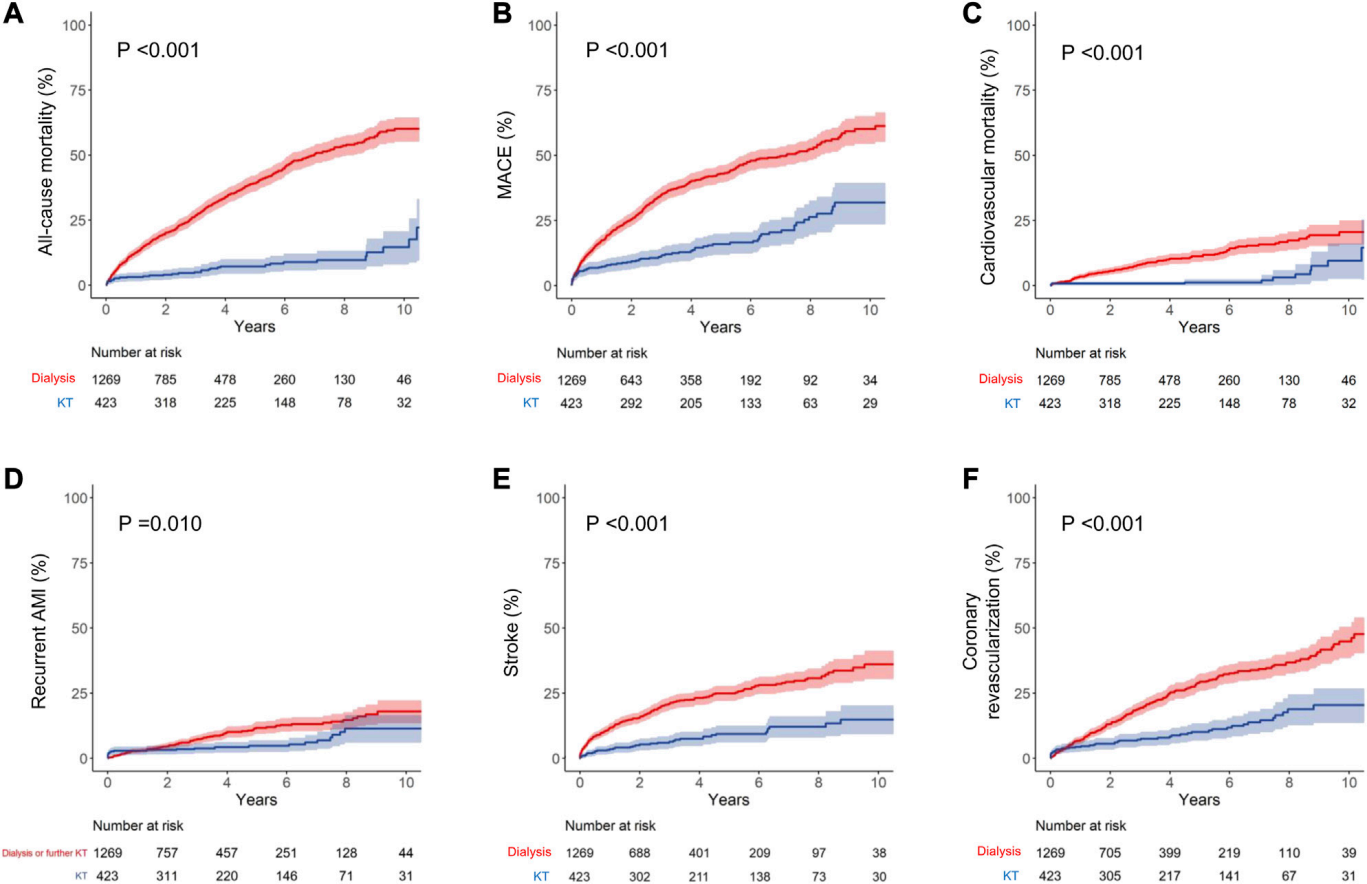
Kaplan–Meier curve analyses for cumulative incidence of each outcome. **(A)** All-cause mortality, **(B)** MACE, **(C)** cardiovascular mortality, **(D)** recurrent AMI, **(E)** stroke, and **(F)** coronary revascularization. Dialysis group data are shown in red and KT group data are shown in blue. MACE is the composite outcome of cardiovascular mortality, non-fatal AMI, and stroke. AMI, acute myocardial infarction; KT, kidney transplantation; MACE, major adverse cardiovascular events.

During the mean follow-up period of 48.3 ± 38.6 months (dialysis group, 45.7 ± 37.7 months; KT group, 61.3 ± 40.7 months), 542 patients in the dialysis group and 41 patients in the KT group died, representing incidence rates of 112.2 and 19.0 per 1,000 person-years, respectively. Except for unknown cause, the most common cause of mortality was CVD, followed by cancer and infection in both groups (Supplementary Figure S4). All-cause mortality was significantly lower in the KT group than in the dialysis group (*p* < 0.001) based on Kaplan–Meier curve analysis (Figure 2). The 1, 5, and 10 years cumulative incidences of all-cause mortality were 12.6%, 39.1%, and 60.1% in the dialysis group and 3.1%, 7.2%, and 14.5% in the KT group (Table 2). The adjusted hazard ratio (HR) of KT for all-cause mortality was 0.17, with a 95% confidence interval (CI) of 0.12–0.24 (*p* < 0.001).

**TABLE 2:** Adjusted hazard ratios of KT for outcomes versus two dialysis control groups.

Outcomes		Cumulative incidence			Fine & gray model			
		1 year	5 years	10 years	Unadjusted sHR (95% CI)	*P*	Adjusted sHR^a^ (95% CI)	*P*
All-cause mortality	Dialysis	12.6	39.1	60.1	Reference		Reference	
	KT	3.1	7.2	14.5	0.17 (0.12–0.24)	<0.001	0.17 (0.12–0.24)	<0.001
MACE^b^	Dialysis	15.6	37.6	52.1	Reference		Reference	
	KT	6.6	13.8	29.1	0.37 (0.28–0.48)	<0.001	0.38 (0.23–0.51)	<0.001
Cardiovascular mortality	Dialysis	3.5	11.3	20.5	Reference		Reference	
	KT	0.7	1.2	9.5	0.22 (0.12–0.41)	<0.001	0.23 (0.12–0.42)	<0.001
Recurrent AMI	Dialysis	2.8	11.7	18.0	Reference		Reference	
	KT	2.9	4.7	11.3	0.56 (0.36–0.87)	0.011	0.59 (0.38–0.93)	0.023
Stroke	Dialysis	11.3	24.5	35.7	Reference		Reference	
	KT	3.5	9.3	14.9	0.34 (0.24–0.48)	<0.001	0.33 (0.23–0.46)	<0.001
Coronary revascularization	Dialysis	6.9	29.3	44.8	Reference		Reference	
	KT	4.4	10.1	20.4	0.38 (0.28–0.52)	<0.001	0.38 (0.27–0.52)	<0.001

For MACE, cardiovascular death, recurrent AMI, stroke, coronary revascularization, other causes of mortality except for CVD, and follow-up loss were considered competing risks. Moreover, regression analyses were performed by Fine and Gray’s model for those outcomes.

Abbreviations: AMI, acute myocardial infarction; CI, confidence intervals; CVD, cardiovascular disease; MACE, major cardiovascular events; KT, kidney transplantation; sHR, subdistribution hazard ratio.

^a^ Adjusted by age, gender, Charlson comorbidity index, interval from AMI to KT or dialysis, and type of AMI treatment. Year of index date.

^b^ MACE means the composite outcome of cardiovascular death, non-fatal AMI, and stroke.

The incidence of MACE was also significantly lower in the KT group than in the dialysis group (*p* < 0.001; Figure 2). The 1, 5, and 10 years cumulative incidences of MACE were 15.6%, 37.6%, and 52.1% in the dialysis group and 6.6%, 13.8%, and 29.1% in the KT group. The adjusted HR of KT for MACE was 0.38, with a 95% CI of 0.23–0.51 (*p* < 0.001; Table 2).

### Secondary Outcomes

The incidences of all MACE components were significantly lower in the KT group than in the matched dialysis controls (Figure 2 and Table 2). KT provided the most protection against cardiovascular death, as indicated by the lowest subdistribution HR (HR, 0.23 [95% CI, 0.12–0.42]; *p* < 0.001). For cardiovascular mortality, the 1, 5, and 10 years cumulative incidences were 3.5%, 11.3%, and 20.5% in the dialysis group and 0.7%, 1.2%, and 9.5% in the KT group. KT was also protective against recurrent AMI (HR, 0.59 [95% CI, 0.38–0.93]; *p* = 0.023) and stroke (HR, 0.33 [95% CI, 0.23–0.46]; *p* < 0.001), compared with maintaining on dialysis. Additionally, the incidence of coronary revascularization (PCI or CABG), regardless of the specific diagnosis, was significantly lower in the KT group than in the dialysis group (HR, 0.38 [95% CI, 0.27–0.52]; *p* < 0.001).

### Sensitivity Analyses

**FIGURE 3:**
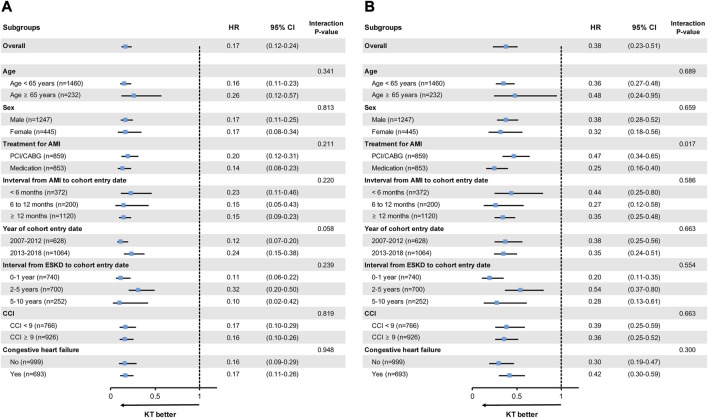
Subgroup analyses for **(A)** all-cause mortality and **(B)** MACE. MACE is the composite outcome of cardiovascular death, non-fatal AMI, and stroke. AMI, acute myocardial infarction; CABG, coronary artery bypass graft; CCI, Charlson Comorbidity Index; CI, confidence interval; ESKD, end-stage kidney disease; HR, hazard ratio; KT, kidney transplantation; MACE, major adverse cardiovascular events; PCI, percutaneous coronary intervention.

The protective effects of KT for all-cause mortality and MACE were seen in all subgroups, especially in higher-risk patients, such as those >65 years of age, patients with an interval from AMI to cohort entry date of <6 months, and those with CHF (Figure 3). However, when compared within the stratified time intervals during the early period after cohort entry, the KT group had a higher risk of recurrent AMI in the first 3 months post-KT, compared with the dialysis group (HR, 3.30 [95% CI, 1.46–7.47]; *p* = 0.004) (Supplementary Tables S3, S4).

## Discussion

To our knowledge, this study is the first population-based cohort study that used nationally representative data to compare all-cause mortality and MACE in patients with ESKD and a prior AMI between patients treated with KT and those maintained on dialysis. KT was associated with a survival benefit in patients with ESKD and an AMI history at certain time points, compared with chronologically matched patients who remained on dialysis at the corresponding time points during the course of their ESKD. Additionally, our results suggested that KT reduced the risk of MACE (overall and all components) in patients with ESKD and a prior AMI, compared with maintenance dialysis. Of the individual MACE components, cardiovascular mortality decreased the most in patients who underwent KT. The beneficial effects of KT for all-cause mortality and MACE were consistent across various subgroups, including patients >65 years, those with a recent (<6 months) AMI, and patients with CHF, all of whom are considered at much higher risk for adverse events following KT. Our results, therefore, suggest that clinicians should actively consider KT for patients with ESKD who have survived a prior AMI.

In previous national cohort studies, the presence of multiple comorbidities was associated with reduced access to KT in patients with ESKD [8–10, 25]. This low access likely reflects clinicians assuming that KT in patients with multiple comorbidities can result in poorer survival than remaining on dialysis. In this regard, studies in Denmark and Argentina demonstrated the clinical relevance of recommending KT, even in patients with multiple comorbidities [13, 26]. However, the survival benefits of KT in patients with ESKD who survived a prior AMI have not been fully investigated. To help fill this knowledge gap, the current study provides evidence in support of the use of KT in patients with a previous AMI.

A major strength of this study was that we compared KT patients with chronologically matched dialysis controls who had similar underlying conditions, including a prior AMI, at similar time points during the course of ESKD. Because most KT patients underwent varying durations of dialysis before transplantation, a standard retrospective study design would inevitably lead to immortal time bias between the initial diagnosis of ESKD and the KT procedure. We minimized potential bias by using a prevalent new user design and matching on time-conditional propensity scores, as has been excellently described by Suissa et al [17–19]. Time-dependent Cox analysis adjusted the hazard ratio by considering time-dependent covariates before and after the reference point at the time of KT. On the other hand, in the case of prevalent new user design, chronological matching was performed to reflect the patient’s status at each time point during each period. This method more accurately aligned the time-dependent coefficients and better reflected the characteristics ESKD patients at a specific time point.

Due to limitations in the data characteristics, we were unable to extract the KT waitlist from NHIS data. While it would be more valid to compare outcomes with wait-listed dialysis patients, it is justified to use all-propensity matched dialysis patients as a control group. Therefore, we established matched controls from the entire pool of dialysis patients in one nation, instead of a waiting-list group of patients. From the perspective of nephrologists and transplant surgeons, waiting-list patients are a specially selected population who are planning to proceed with KT, regardless of donor type. Waiting-list analysis is suitable for investigating the benefits of a specific type of KT, such as lymphocyte cross match [27] or ABO-incompatible living-donor KT [28]; however, it cannot help decide whether to proceed with KT (i.e., begin waiting for a deceased donor or undergo living-donor KT) in patients with ESKD and multiple comorbidities, whose access to transplantation would be low. Thus, our study was designed to compare patients who underwent KT with those maintained on dialysis at the corresponding date, regardless of whether they were waitlisted or received a transplant at a later date. To clarify the impact on outcome, disease entity was restricted to AMI, maintaining disease homogeneity. Our findings showed the superiority of KT over dialysis, even for patients with an AMI history, at specific time points after ESKD diagnosis.

A prior study using the US Renal Data System showed that the cumulative incidence of AMI in patients who underwent KT, regardless of whether they received a deceased donor and living-donor kidney, was higher than that of patients on dialysis maintenance until approximately 1 year after KT [29]. Over time, the incidence of AMI in patients who underwent KT eventually became lower than that in patients on maintenance dialysis. Indeed, KT patients have several risk factors for MACE, especially during the early post-transplantation period, such as the stress of surgery, the high dose of immunosuppressive medications, and the possibility of early graft dysfunction [30]. In our study, recurrent AMI in KT patients with an AMI history was also significantly higher than that of dialysis patients in the first 3 months after cohort entry. Given that the overall incidence of recurrent AMI, as well as other MACE components, can be reduced by KT, the risk of recurrent AMI during the early post-transplantation period should not be the reason for automatically avoiding KT in this higher-risk ESKD population. However, clinicians should be cautious about the possibility of early recurrent AMI and monitor patients closely to allow prompt detection of this event.

Given that intravenous contrast and CABG surgery negatively affect residual renal function, patients with ESKD (including those receiving or not receiving dialysis) are less likely to undergo diagnostic coronary angiography or coronary revascularization after AMI [24]. In our study, half of the patients with ESKD and an AMI history did not undergo CABG or PCI and received only medical treatment. For patients with chronic kidney disease, guidelines recommend standard treatment, regardless of renal function, in the setting of ST-elevation MI; however, in the setting of non-ST-elevation MI, there is insufficient evidence to recommend standard therapy, especially for patients with ESKD [31, 32]. It is difficult to say which treatment is superior for patients with ESKD and an AMI history because prognosis varies depending on the individual circumstances, such as the presence of left anterior descending coronary artery disease [33, 34]. However, regardless of the type of prior AMI treatment (PCI, CABG, or medications alone), subsequent KT showed a survival benefit in our study population.

Dual antiplatelet therapy is usually required for 6 months to 1 year after coronary revascularization by either PCI or CABG [35]. Therefore, in the early post-revascularization period, especially <6 months post-AMI, clinicians are likely reluctant to suggest KT because of the possibility of MACE recurrence or bleeding secondary to antiplatelet therapy. However, our results indicated that KT could be beneficial, even before 6 months after an AMI. Furthermore, the duration and number of antiplatelet agents could be minimized through appropriate stent selection or CABG, thereby reducing the interval from coronary revascularization to KT [36]. In a study from the United Kingdom, of patients who underwent pre-KT assessment with coronary angiography, most revascularization procedures before KT were successful, and the 3 years survival of patients after cardiac revascularization was 88.4% [37]. Considering this report and our results, we suggest that planned KT after a minimized interval with antiplatelet treatment is feasible when patients with ESKD develop AMI.

CHF is closely associated with the general health status of patients with ESKD [38]. When considering KT, CHF is an important factor for determining how well patients tolerate the operation and negatively impacts the likelihood of a clinician considering KT. However, our study showed that among AMI survivors, subgroup with CHF also had a survival benefit from KT. This result provides evidence for more actively planning KT, even in patients with a prior AMI and CHF. However, because information about ejection fraction and New York Heart Failure Association (NYHA) classification was not available for this study, this result should be interpreted with caution.

This study has several limitations. Despite successful matching, we could not completely eliminate selection bias between the KT and dialysis groups because of limited information in the claims database, such as laboratory results, severity of prior AMI, and NYHA functional classes. Another limitation was the lack of information about time-varying CVD risk factors, such as diet, physical activity, and medications during follow-up. We also could not distinguish donor characteristics, such as living or deceased, age, renal function at donation, and underlying disease, all of which are important factors affecting post-transplantation outcomes. Lastly, we could not estimate the likelihood of undergoing KT (especially deceased donor KT after being waitlisted) because the NHIS database does not contain information about the blood group or degree of pre-transplantation sensitization of KT patients.

Despite these limitations, the results of this nationwide population-based cohort study showed that KT was associated with lower all-cause mortality and MACE in patients with ESKD and an AMI history, even in various high-risk subgroups. Thus, KT seems safe among AMI survivors who are planning to receive dialysis or are currently on dialysis, unless another definite contraindication is present.

## Data Availability

The database used for this study was provided by the National Health Insurance Service (NHIS) in the Republic of Korea (NHIS-2019-1-448). Only authorized researchers were granted access to the database at the Big Data Research Center of the Big Data Steering Department at the NHIS.

## Footnotes

1
http://mdis.kostat.go.kr

2
http://nhiss.nhis.or.kr

3
http://cran.r-progect.org/

## Abbreviations

AMI, acute myocardial infarction; CABG, coronary artery bypass graft; CHF, congestive heart failure; CVD, cardiovascular disease; ESKD, end stage kidney disease; KT, kidney transplantation; MACE, major adverse cardiovascular events; PCI, percutaneous coronary intervention.
