# Supplementary material for: Survival Benefit of Kidney Transplantation in Patients With End-Stage Kidney Disease and Prior Acute Myocardial Infarction
Source: Transpl Int. 2023 Aug 24;36:11491. doi: 10.3389/ti.2023.11491 (PMC10483068; doi:10.3389/ti.2023.11491)

Supplementary materials

Supplementary Table S1. Diagnosis, procedure, and specific codes.

Disease	Diagnosis, procedure, specific codes
Acute myocardial infarction	I21, I22, I23
Cancer	C00-C97
Percutaneous coronary intervention	M655, M656, M657, M663
Coronary artery bypass graft	O164, O183, OA64
Conduction abnormality	I441, I442, I495, Z950
Stroke	I60-63 and admitted more than two days
Diabetes	E11-14 at least one antidiabetic medication
Hemodialysis	Z491, N185, I120, Z992, O7020, O7021, V001
Peritoneal dialysis	Z492, O7061, O7062, O7071, O7072, O7074, O7076, O7077, V003
Kidney transplantation	R3280, V005
Valve disorder	I05-I08, I34-37, Z952-Z954
Vascular disease	I20, I21, I22, I24, I25

All of the diagnostic, procedure, specific codes are shown in https://www.hira.or.kr/

Supplementary Table S2. Drugs (code and dose) used for National Health Insurance Korea database analysis.

Drug	HIRA code
ACEIs or ARBs or aliskiren	104201ATB, 104202ATB, 114701ATB, 122601ATB, 122602ATB, 122603ATB, 122901ATB, 122902ATB, 122903ATB, 133001ATB, 133002ATB, 133003ATB, 140901ATB, 140902ATB, 151601ATB, 151603ATB, 163501ATB, 163502ATB, 104201ATB, 104202ATB,114701ATB, 122601ATB, 122602ATB, 122603ATB, 122901ATB, 122902ATB, 122903ATB, 133001ATB, 133002ATB, 133003ATB, 140901ATB, 140902ATB, 151601ATB, 151603ATB, 163501ATB, 163502ATB, 173401ATB, 173402ATB, 177301ATB, 177303ATB, , 177303ATB, 184501ATB, 185701ATB, 185702ATB, 196801ATB, 196802ATB, 211301ATB, 211302ATB, 221901ATB, 222401ACH, 222401ATB, 222402ACH, 222402ATB, 222404ATB, 235002ATB, 247101ATB, 247102ATB, 247103ATB, 247104ATB, 378801ATB, 378802ATB, 378803ATB, 429201ATB, 468501ATB, 468502ATB, 468503ATB, 501601ATB, 501602ATB, 510401ATB, 510402ATB, 510403ATB, 515201ATB, 515202ATB, 515203ATB, 520901ATB, 520902ATB, 662401ATB, 662402ATB, 662403ATB, 651401ATB, 651402ATB, 651403ATB
BBs	117903ATB, 117904ATB, 124801ATB, 219901ATB, 219902BIJ, 219904ATB, 219905ACR, 219906ACR, 111401ATB, 111402ATB, 111403ATB, 116801ATB, 116803ATB, 117001ATB, 117002ATB, 125001ATB, 125002ATB, 125003ATB, 125004ACR, 125005ATB, 125006ACR, 125007ACR, 125007ATR, 125008ACR, 125008ATR, 483101ATB, 483102ATB, 489501ATB, 489502ATB, 489503ATB, 662201ATB, 662202ATB, 117901ATB, 117902ATB, 129101ATB, 193802ATB, 194003ATR, 198301ATB, 154401BIJ, 154402BIJ, 154431BIJ, 154430BIJ, 180201ATB, 180201BIJ, 180202BIJ, 180230BIJ, 180231BIJ
CCBs	107601ATB, 107601ATD, 107602ATB, 107602ATD, 107603ATB, 114001ACH, 114002ACH, 114003ACH, 115101ATB, 115102ATB, 115103ATB, 115104ATB, 133101ATB, 133102ATB, 157501ATR, 157503ATR, 178902ACR, 180301ATB, 180302ATB, 180303ATB, 182001ATB, 182002ATB, 188001ATB, 188002ATB, 201001BIJ, 201002ATB, 201002BIJ, 201003ACR, 201030BIJ, 201031BIJ, 201033BIJ, 202401ATB, 202402ACS, 202402ATB, 247601ACR, 247603ATR, 247604BIJ, 247605ATR, 247606ATB, 247607ATB, 459801ACH, 459801ATB, 459802ACH, 459901ATB, 459902ATB, 464601ATB, 470801ATB, 470802ATB, 476201ATB, 479701ATB, 483201ATB, 483202ATB, 489501ATB, 489502ATB, 489503ATB, 145702BIJ, 145703ACR, 145704BIJ, 145706ATB, 145706ATR, 145707ACR, 145707ATB, 145707ATR, 201401ACS, 201401ATB, 201402ATB, 201405ATR, 201407ACS, 201408ATR, 201409ATR, 201702ATB, 201901ATB, 201902BIJ, 201930BIJ, 356201ATB, 356202ATB, 356202ATR, 356203ATR, 441201ATB, 441202ATB, 528201ATR, 528202ATR, 145702BIJ, 247630BIJ, 486501ATB, 486502ATB, 495901ATB, 501701ATB

Diuretics	101501ATB, 101502BIJ, 106901ATB, 163801ATB, 163802BIJ, 163830BIJ, 170801ATB, 174401ATR, 174402ATB, 174403ATB, 231101ATB, 231102ATB, 244701ATB, 262700ATB, 367001ATB, 367002ATB, 451301ATB, 451302ATB
Vasodilators	170701ATB, 170702BIJ, 196102ATB, 471401CSI, 471430CSI, 485201ATB, 485202ATB, 553301ATB, 564701ATB, 564702ATB, 632201ATB, 652301ATB, 652302ATB, 652303ATB, 170730BIJ, 512401BIJ, 512402BIJ, 512403BIJ, 512430BIJ, 512431BIJ, 512432BIJ,
ACEIs or ARBs and CCB	447100ATB, 447200ATB, 466000ATB, 492800ATB, 492900ATB, 495800ATB, 500500ATB, 500600ATB, 582200ATB, 582400ATB, 502700ATB, 503000ATB, 513900ATB, 511500ATB, 511600ATB, 511700ATB, 623100ATB, 521200ATB, 521300ATB, 521400ATB, 644800ATB, 522200ATB, 522300ATB, 522400ATB, 522600ATB, 522700ATB, 522800ATB, 522900ATB, 523000ATB, 523100ATB, 523200ATB, 523300ATB, 523400ATB, 547500ATB, 547600ATB, 547700ATB, 547800ATB, 547900ATB, 548000ATB, 631300ATB, 629400ATB, 629500ATB, 629600ATB, 632800ATB, 632900ATB, 633000ATB, 637400ATB, 637500ATB, 637600ATB, 644800ATB, 651900ATB, 652000ATB, 652100ATB, 652700ATB, 652900ATB, 653000ATB, 653100ATB
ACEIs or ARBs and statin	524000ATB, 524100ATB, 527000ATB, 527100ATB, 525000ATB, 525100ATB, 525200ATB, 525300ATB, 629700ATB, 629800ATB, 526300ATB, 526400ATB, 526500ATB, 526900ATB, 644100ATB, 644200ATB, 653200ATB, 629900ATB, 630000ATB, 630100ATB, 630200ATB, 631600ATB, 631700ATB, 634900ATB, 635000ATB, 635100ATB, 635200ATB, 653200ATB, 654600ATB, 654700ATB, 654800ATB, 654900ATB, 655000ATB, 661800ATB, 661900ATB, 662000ATB, 662100ATB, 673700ATB, 688100ATB, 688200ATB, 688300ATB, 688400ATB, 688500ATB
BB with diuretics	262100ATB, 262600ATB, 460200ATB, 469800ATB, 469900ATB, 470000ATB
ACEIs or ARBs and diuretics	262200ATB, 262300ATB, 262500ATB, 378900ATB, 440300ATB, 453600ATB, 453700ATB, 486900ATB, 356400ATB, 442600ATB, 385700ATB, 385800ATB, 423700ATB, 440800ATB, 443200ATB, 443300ATB, 502600ATB, 448600ATB, 448700ATB, 460500ATB, 477400ATB, 490100ATB, 497900ATB, 499200ATB, 499300ATB, 513600ATB, 522000ATB, 526800ATB, 556200ATB, 673500ATB, 673600ATB
ACEIs or ARBs and CCB and diuretics	519700ATB, 519800ATB, 519900ATB, 520000ATB, 520100ATB, 662800ATB, 662900ATB, 663000ATB, 663500ATB, 663600ATB, 663700ATB, 663800ATB, 682700ATB, 682800ATB, 682900ATB
ACEIs or ARBs and CCB and statin	663900ATB, 664000ATB, 664100ATB, 664200ATB, 664300ATB, 664400ATB, 671200ATB, 671300ATB, 671400ATB, 671500ATB, 671600ATB, 671700ATB, 677000ATB, 677100ATB, 677300ATB, 677400ATB, 677500ATB, 677600ATB, 686800ATB, 679500ATB, 679600ATB, 679700ATB, 680300ATB, 684300ATB, 684400ATB, 684500ATB, 684600ATB, 684700ATB, 686800ATB, 686900ATB, 690400ATB, 690500ATB, 690600ATB, 690700ATB, 691400ATB, 691500ATB

CCB and statin	472300ATB, 472400ATB, 472500ATB, 518900ATB, 614500ATB, 673900ATB, 674000ATB, 674100ATB, 678600ATB
BB and CCB	262400ATR
BB and statin	683000ATB, 683100ATB, 683200ATB, 691200ATB
Alpha blocker	149101ATB, 149102ATB, 149104ATR, 483401ACH, 104803ATR, 159001ATB, 234601ACR, 234601ATD, 234601ATR, 234602ACR, 234603ACR, 234603ATD, 234603ATR, 235501ATB, 235502ATB, 235503ATB, 458801ACS, 458801ATB, 504201ACH, 504202ACH, 504202ATB, 504203ACH, 504203ATD, 505801ATB, 505802ATD, 614201ATB, 614202ATB, 614203ATB
Statin or ezetimibe or fibrate	111501ATB, 111502ATB, 111503ATB, 111504ATB, 162401ACH, 162402ACH, 162403ATR, 185801ATB, 216601ATB, 216602ATB, 216603ATB, 216604ATB, 218001ATB, 227801ATB, 227801ATR, 227802ATB, 227803ATB, 227805ATB, 227806ATB, 454001ATB, 454002ATB, 454003ATB, 462201ATB, 470901ATB, 470902ATB, 470903ATB, 471000ATB, 471100ATB, 507800ATB, 502201ATB, 502202ATB, 502203ATB, 502204ATB, 519300ACH, 631400ATB, 631500ATB, 633800ATB, 633900ATB, 634600ATB, 634800ATB, 640700ATB, 640800ATB, 640900ATB, 663400ACS, 679300ACH
DM medication	170101BIJ, 170102BIJ, 170103BIJ, 170130BIJ, 170131BIJ, 170401BIJ, 170402BIJ, 170430BIJ, 170431BIJ, 170502BIJ, 175301BIJ, 175302BIJ, 175304BIJ, 175330BIJ, 175331BIJ, 175332BIJ, 175333BIJ, 441301BIJ, 441302BIJ, 441303BIJ, 441304BIJ, 441305BIJ, 441330BIJ, 441331BIJ, 441332BIJ, 441333BIJ, 441334BIJ, 461801BIJ, 461802BIJ, 461804BIJ, 461830BIJ, 461831BIJ, 461832BIJ, 484901BIJ, 484902BIJ,484930BIJ, 484931BIJ, 488701BIJ, 488730BIJ, 507401BIJ, 626700BIJ, 626801BIJ, 626802BIJ, 626830BIJ, 626831BIJ, 512101BIJ, 512102BIJ, 512130BIJ, 512131BIJ, 626601BIJ, 626602BIJ, 626630BIJ, 626631BIJ, 639701BIJ, 639702BIJ, 644501BIJ, 644502BIJ, 666700BIJ, 667000BIJ, 527301ATB, 527302ATB, 628201ATB, 628202ATB, 636101ATB, 639800ATR, 641400ATR, 649000ATB, 649100ATB, 649200ATB, 649300ATB, 649400ATB, 649500ATB, 674301ATB, 674302ATB, 100601ATB, 100602ATB, 165402ATB, 165601ACS, 165602ACS, 165602ATB, 165603ATR, 165604ATR, 165701ATB, 165702ATB, 165703ATB, 165704ATB, 165801ATB, 191501ATB, 191502AGR, 191502ATB, 191502ATR, 191503ATB, 191504ATB, 191504ATR, 191505ATR, 249001ATB, 249002ATB, 249002ATD, 348002ATB, 379501ATB, 379502ATB, 379503ATB, 406201ATB, 406202ATB, 421100ATB, 430201ATB, 430202ATB, 430203ATB, 431901ATB, 431902ATB, 443400ATB, 443500ATB, 452700ATB, 452900ATB, 469100ATB, 471900ATB, 474200ATB, 474300ATB, 474300ATR, 488800ATB, 488900ATB, 489000ATB, 498100ATB, 498600ATB, 486101ATB, 497200ATB, 498100ATB, 523600ATB, 523700ATB, 525500ATB, 525600ATB, 525901ATB, 631900ATB, 632100ATB, 637200ATB, 653800ATR, 653900ATR, 654000ATR, 655700ATR, 518800ATB, 500801ATB, 501101ATB, 501102ATB, 501103ATB, 502200ATB, 502300ATB, 502300ATR, 502900ATB, 513700ATB, 513700ATR, 524700ATR,

	507000ATB, 507100ATB, 519600ATB, 518500ATR, 518600ATR, 520500ATB, 520600ATB, 520700ATB, 523800ATR, 632000ATR, 645000ATR, 654100ATR, 613301ATB, 613302ATB, 616401ATB, 619101ATB, 624201ATB, 624202ATB, 624203ATB, 627301ATB, 630300ATB, 630400ATB, 630500ATB, 630600ATB, 635600ATB, 635700ATB, 675500ATB, 639601ATB, 641800ATR, 641900ATR, 642000ATR, 645301ATB, 648400ATB, 648500ATB, 648600ATB, 649900ATR, 650000ATR, 650100ATR
DM medication and statin	664600ATB, 664700ATB, 664800ATB, 671800ATR, 673800ATR, 671900ATR, 672000ATR, 672100ATR, 672500ATR, 672600ATR, 672700ATR, 672800ATR, 672900ATR, 673000ATR, 683300ATR, 683400ATR
Antiplatelet (aspirin or clopidogrel or aspirin and clopidogrel or cilostazole or ticlopidine or prasugrel or ticagrelor or triflusal)	110701ATB, 110702ATB, 110801ATB, 110802ATB, 111001ACE, 111001ACH, 111001ATB, 111001ATE, 111002ATE, 111003ACE, 111003ATE, 136901ATB, 492501ATB, 495201ATB, 498801ATB, 501501ATB, 517900ACE, 517900ACH, 517900ATE, 667500ACE, 133201ACR, 133201ATB, 133201ATR, 133202ATB, 133203ACR, 133203ATR, 506100ATB, 687200ATR, 498900ATB, 597301ATB, 597302ATB, 615901ATB, 615902ATB, 244101ACH, 244102ACH

Abbreviations: ACEi, angiotensin converting enzyme inhibitors; ARBs; angiotensin receptor blockers; BB, beta blockers; CCBs, calcium channel blockers; DM, diabetes mellitus

Supplementary Table S3. Cox regression for outcomes within stratified time intervals during early period after cohort entry date

Variables	Group	Cumulative incidence (%)			Before 3 months			Before 6 months			Before 12 months		
		3 months	6 months	12 months	HR	95% CI	*P*-value	HR	95% CI	*P*-value	HR	95% CI	*P*-value
All-cause mortality	ESKD	4.41	7.97	12.55	1			1			1		
	KT	1.90	2.86	3.12	0.42	0.20-0.88	0.021	0.35	0.19-0.64	0.001	0.25	0.14-0.43	<0.001
MACE	ESKD	7.23	11.07	15.62	1			1			1		
	KT	5.00	5.50	6.55	0.69	0.43-1.11	0.126	0.49	0.32-0.77	0.002	0.41	0.27-0.61	<0.001
CVD mortality	ESKD	1.12	1.54	3.51	1			1			1		
	KT	0.71	0.71	0.71	0.64	0.18-2.23	0.486	0.47	0.14-1.60	0.227	0.22	0.07-0.70	0.011
Recurrent AMI	ESKD	0.89	1.65	2.81	1			1			1		
	KT	2.85	2.85	3.14	3.3	1.46-7.47	0.004	1.81	0.88-3.70	0.105	1.09	0.56-2.11	0.795
Stroke	ESKD	5.33	8.03	11.07	1			1			1		
	KT	1.93	2.44	3.50	0.35	0.17-0.74	0.006	0.3	0.15-0.57	<0.001	0.3	0.17-0.52	<0.001
Revascularization	ESKD	2.34	3.77	6.89	1			1			1		
	KT	3.33	3.83	4.36	1.45	0.77-2.75	0.25	1.04	0.59-1.84	0.886	0.66	0.40-1.10	0.111

Abbreviations: AMI, acute myocardial infarction; CVD, cardiovascular disease; ESKD, end stage kidney disease; MACE, major adverse cardiovascular event; KT, kidney transplantation

Supplementary Table S4. The raw number of event each outcomes

Variables	Group	Number of events		
		3 months	6 months	12 months
All-cause mortality	ESKD	45	83	129
	KT	9	13	14
MACE	ESKD	60	97	153
	KT	18	21	25
CVD mortality	ESKD	14	20	40
	KT	4	4	4
Recurrent AMI	ESKD	14	26	52
	KT	11	12	12
Stroke	ESKD	46	72	103
	KT	8	10	14
Revascularization	ESKD	25	42	74
	KT	11	13	15

Abbreviations: AMI, acute myocardial infarction; CVD, cardiovascular disease; ESKD, end stage kidney disease; MACE, major adverse cardiovascular event; KT, kidney transplantation

Supplementary Figures
Supplementary Fig. S1. Graphic depiction of establishing time-based cohort.

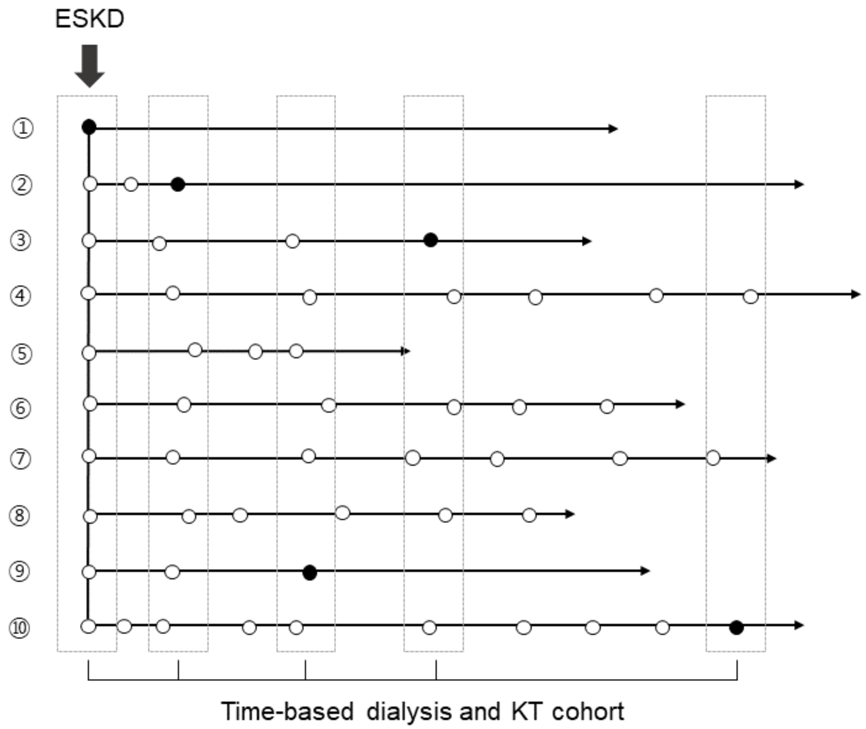

White round mark means prescription of dialysis and black round mark means KT. Time-based cohorts were established with time interval of ± 3 month surrounding KT date.

Supplementary Fig. S2. Balanced test before and after propensity score matching between the KT and dialysis groups.

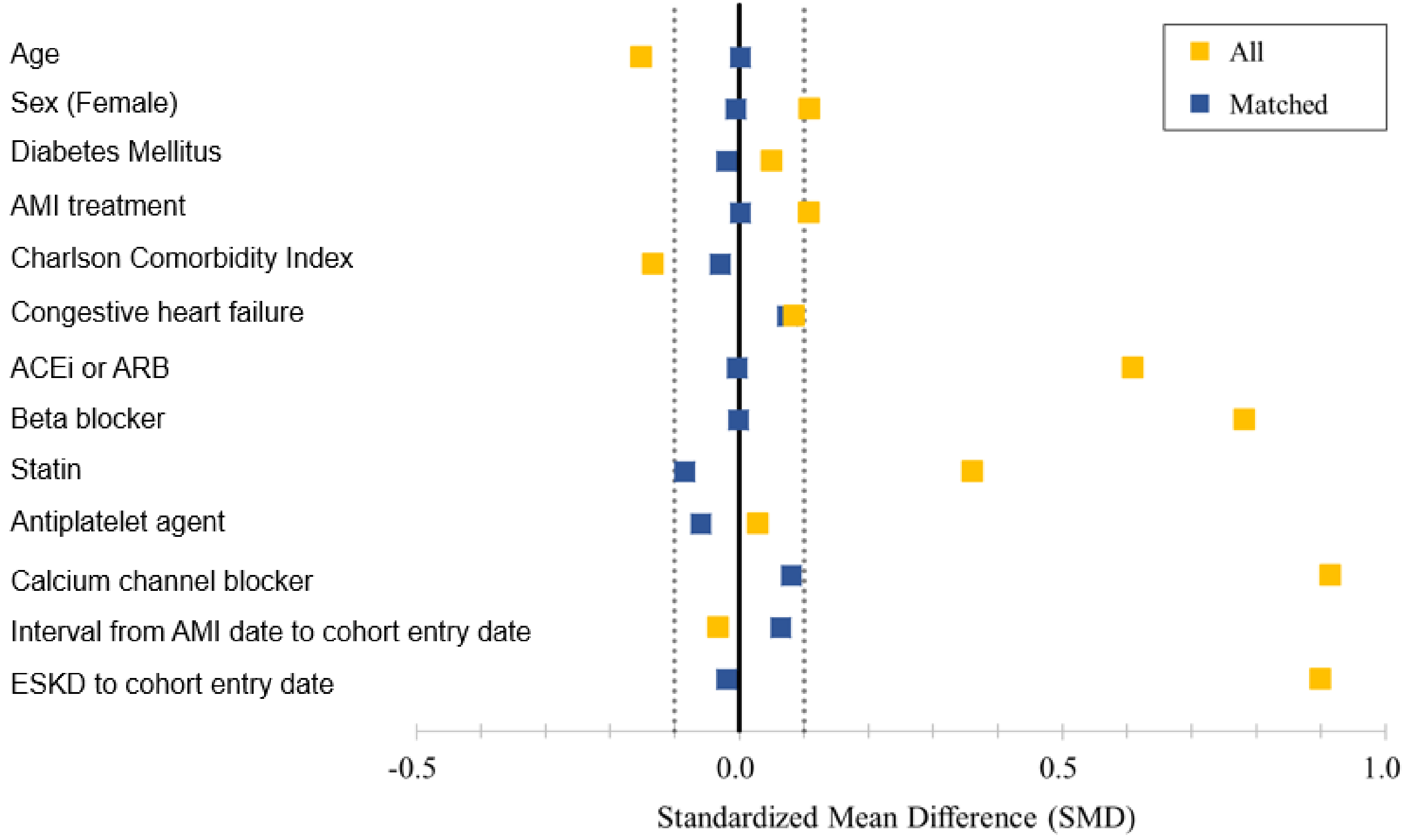

Absolute standardized mean difference before (yellow) and after matching (blue)
Abbreviations: ACEi, angiotensin converting enzyme inhibitors; AMI, acute myocardial infarction; ARB, angiotensin receptor blocker; ESKD, end stage kidney disease

Supplementary Fig. S3. Cumulative incidence of graft failure (restart of dialysis or re-transplantation) in the KT group.

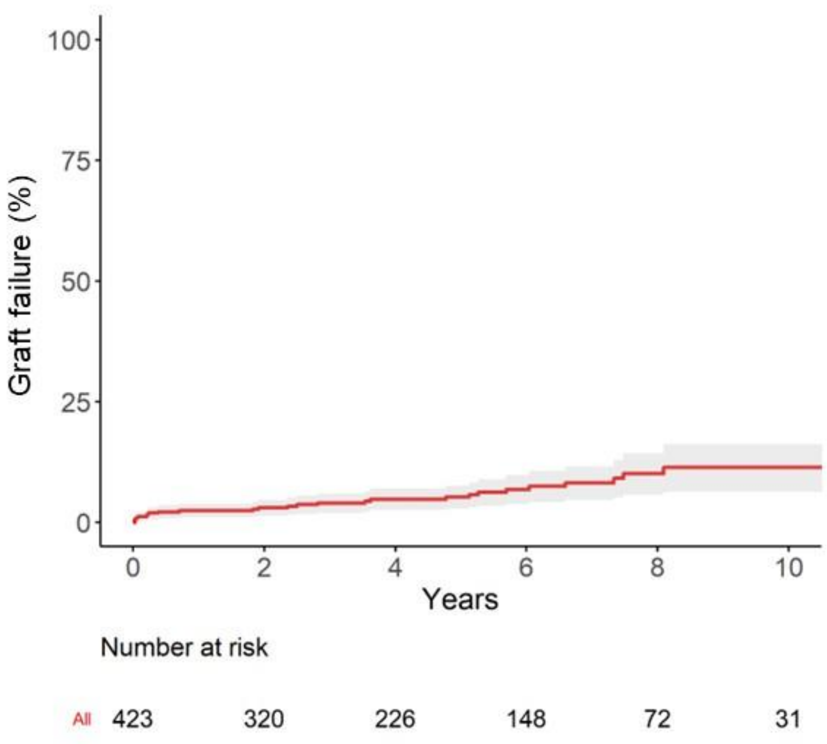

Supplementary Fig. S4. Cause of death for (a) dialysis group and (b) KT group including unknown cause.

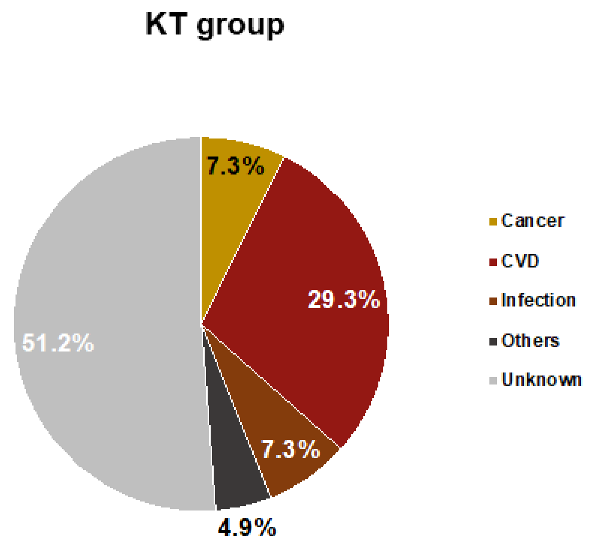

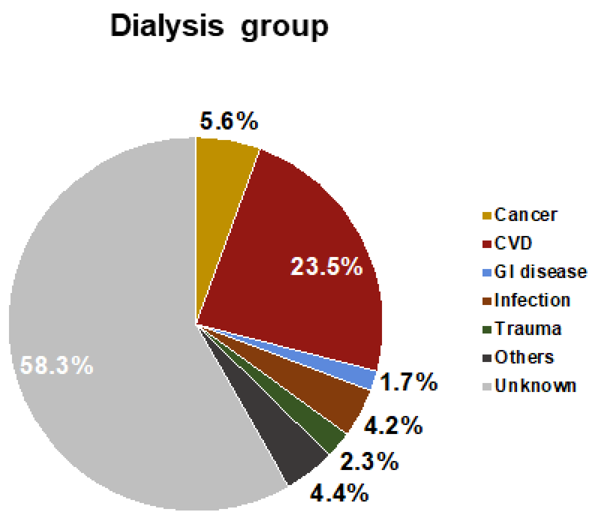

Supplement: Supplementary file 1 [file DataSheet1.pdf]
